# Ideal Illumination for Smartphone-based Trabeculectomy Bleb Photography

**DOI:** 10.18502/jovr.v16i3.9432

**Published:** 2021-07-29

**Authors:** Gagan Kalra, Parul Ichhpujani, Sahil Thakur, Urvashi Sharma

**Affiliations:** ^1^Department of Ophthalmology, Government Medical College and Hospital, Sector-32, Chandigarh, India; ^2^Department of Ocular Epidemiology, Singapore Eye Research Institute, Singapore

**Keywords:** Bleb, Smartphone Photography, Teleophthalmology, Trabeculectomy

## Abstract

**Purpose:**

Ophthalmology has seen numerous novel uses for smartphones over the years including fundus photography, telemedicine, and operative videography. However, anterior segment photography for assessing and documenting trabeculectomy bleb morphology using a smartphone has not been explored in detail. With the current study, we aim to characterize ideal illumination for the anterior segment smartphone photography in trabeculectomy patients.

**Methods:**

Thirty status post-trabeculectomy patients were enrolled in this study. Native camera application and FiLMiC pro camera application were used on iPhone X to compare bleb images using yellow and white pen-torches as illumination source. Measured bleb area was compared using ImageJ software from the two apps in different illumination settings by charting boxplots and using one-way ANOVA test using R software to establish consistency. Bland-Altman interoperability for repeatability of bleb-area measurements was analyzed by plotting Bland-Altman plots. Signal-to-noise ratio was calculated using ImageJ for native camera images using slit-lamp camera images as reference. Subjective rating of these images was then performed by two experienced ophthalmologists and kappa coefficient was calculated for inter-operator repeatability. Statistical analysis was performed.

**Results:**

The measured bleb area from images taken from both apps showed no significant difference, thereby establishing consistency, and Bland-Altman analysis indicated good repeatability and reproducibility. It was noted that SNR was lower for images shot in close illumination as compared to the ones shot in intermediate and distant illumination. Cohen's kappa coefficient was 0.7 for images with distant illumination using white light and 0.65 for images clicked with illumination at an intermediate distance using yellow light, suggesting substantial agreement between the observers.

**Conclusion:**

Smartphone photography is a reliable tool for morphological assessment trabeculectomy blebs. Optimal illumination helps achieve results free from digital noise and better delineation of specific morphological features. Intermediate illumination and distant illumination provides much better results in terms of high SNR while avoiding overexposure and clipping of highlight information in the images.

##  INTRODUCTION

Recent technology and advances in optics of smartphones has persuaded ophthalmologists to use smartphones for numerous novel purposes including fundus photography, telemedicine, operative videography, ophthalmological teaching, training, ophthalmic screening in an emergency setting, etc.^[1,2,3,4]^ The novelty of using smartphones in ophthalmic practice lies in the widespread availability, affordability, and network accessibility that they bring to the table.^[5,6]^ These applications have the ability to decentralize expert care from a tertiary care center and make it accessible to primary care peripheral centers by the means of tele-ophthalmology.^[7]^ There are multiple studies that demonstrate the effectiveness and safety of smartphones for ophthalmological applications.^[5,6][8]^ However, anterior segment photography for assessing and documenting trabeculectomy bleb morphology using a smartphone has not been explored in much detail. Currently, there are no objective guidelines directing appropriate illumination parameters for the ocular surface for better smartphone-based trabeculectomy bleb morphology assessment. In the current study, we attempt to characterize the same.

##  METHODS

This pilot observational study compared the impact of alteration in illumination in terms of distance from ocular surface and color of light source on iPhone X-assisted trabeculectomy bleb photography. Thirty patients who had undergone trabeculectomy for refractory primary open or angle closure glaucoma and registered in Glaucoma Clinic of Department of Ophthalmology, Government Medical College and Hospital, Chandigarh, India were enrolled in the study after obtaining written, informed consent in their vernacular language. This study conforms to the tenets of Declaration of Helsinki.

### Equipment

The native iOS camera app with automatic focusing exposure control was used and the results were compared with those with a third-party camera app – FiLMiC Pro (Cinegenix LLC, Seattle, WA, USA; http://filmicpro.com/) that allowed precise manual control for parameters like focus assist, ISO, and shutter speed. We chose FiLMiC Pro app as it has been previously validated for use in ocular photography.^[1,6,9]^ A 5-watt yellow LED pen-torch and a white LED pen-torch (with single fresh AAA battery) were used. By increasing the distance between the light source and the ocular surface, illumination was changed from close (4 cm) to intermediate (7 cm) to distant illumination (12 cm), in order to evaluate the impact of light source distance on image quality. Our null hypothesis was that there would be no significant difference in the bleb area measured on smartphone imaging with variation in light color and illumination distance. Lux Light Meter Pro app (Elena Polyanskaya) for iPhone was used to quantify the changes in the light intensity (in foot-candles [FC]) at the ocular surface for each scenario. A 15-cm surgical ruler (VISCOT Medical, LLC) held by an assistant was used to measure all distances from the outer canthus of the patient's eye as a landmark to standardize the distances in this study.

### Smartphone Photography

**Figure 1:**
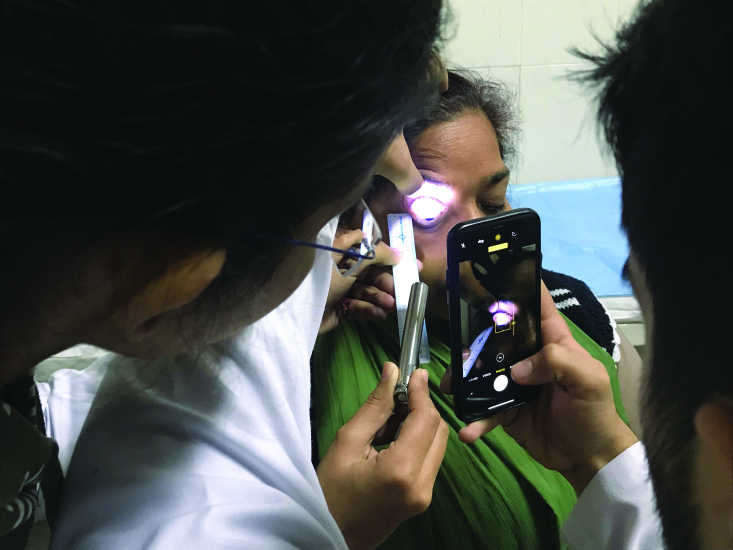
Bleb image being captured using smartphone.

Each patient underwent smartphone photography (same photographer, GK) in the same examination room with a fluorescent tube-light (luminous flux: 2500 lm; power 36W; lamp current: 0.44A; 103V) facing patient's back. The details of the session were discussed with the patient in order to avoid any menace reflex. The room had no windows to avoid any optical interference. As examiner retracted the upper eyelid, the patient was asked to fix gaze in downward direction in order to minimize discomfort from the light source. Initially, three illumination readings were taken at the superior bulbar conjunctiva without any additional light source using the Lux Light Meter Pro app by keeping the iPhone steady at a fixed distance (focusing distance) in front of the eye, and the mean of three readings was calculated to account for the ambient light in the examination room and that came out to be 21.5 lux. An assistant (US) then held a pen torch with yellow light against the measuring scale placed at the outer canthus of the eye [Figure 1] at close, intermediate, and distant illumination sequentially, and sets of three illumination readings (in FC) were obtained from the ocular surface using the Lux Light Meter Pro app. Images were then obtained from iPhone X in the three lighting scenarios using no attachment and keeping the iPhone steady at the minimum focusing distance from the ocular surface. Subsequently, the exercise was repeated using a pen torch with white light.

**Figure 2:**
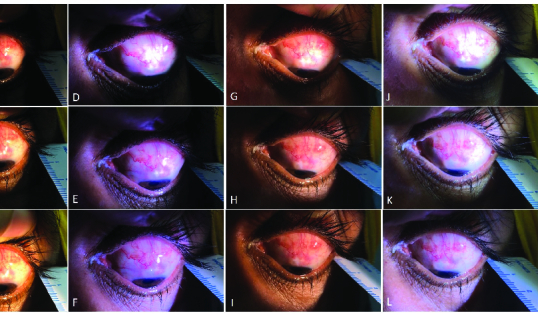
Native camera app images: (A) Close, (B) Intermediate, (C) Distant with yellow pen torch; (D) Close, (E) Intermediate, (F) Distant with white pen torch; FiLMiC Pro app images: (G) Close, (H) Intermediate, (I) Distant with yellow pen torch; (J) Close, (K) Intermediate, (L) Distant with white pen torch.

**Figure 3:**
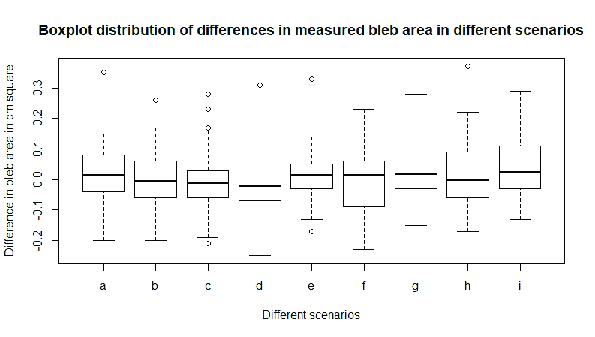
Box-plots for differences in measured bleb area in six different lighting scenarios for native camera application: (A) White-light close filmic vs yellow-light close filmic; (B) White-light close native vs yellow-light close native; (C) White-light close filmic vs yellow-light close native; (D) White-light intermediate filmic vs yellow-light intermediate filmic; (E) White-light intermediate native vs yellow-light intermediate native; (F) White-light intermediate filmic vs yellow-light intermediate native; (G) White-light distant filmic vs yellow-light distant filmic; (H) White-light distant native vs yellow-light distant native; and (I) White-light distant filmic vs yellow-light distant native.

**Figure 4:**
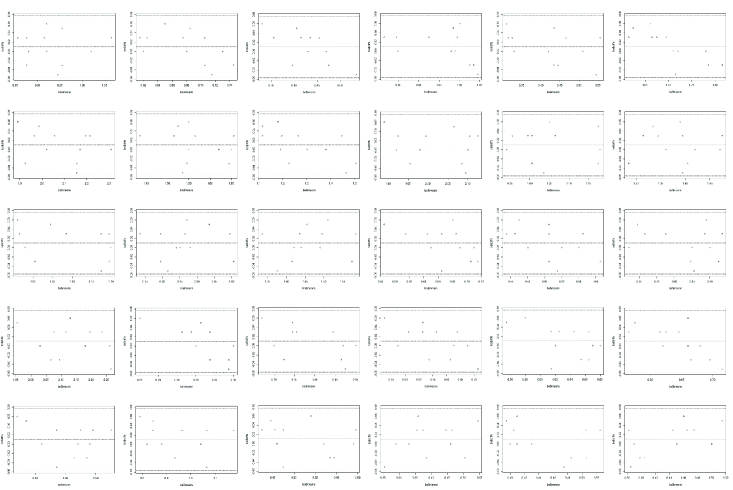
**Supplementary data:** Bland-Altman plots for bleb area measurement between two graders for all eyes included in this study under different lighting scenarios.

### Image Processing

The images obtained from the native app were shot with the iPhone X (1 × ) optical lens at 12.0 MP (4000 × 3000 pixels) resolution with focusing and exposure locked by pressing and holding on the area of interest and then dragging down to adjust exposure. Images were obtained as high-quality screen captures from the 4k (3840 × 2160 pixels) 60 fps video recordings from the FiLMiC Pro app on the iPhone X. For the Filmic Pro video recording, grades analyzed both the unedited video and the still frames. Audio was removed from the video recording to eliminate bias.

Mean of the three FC readings obtained by the exercise was corrected for ambient lighting by subtracting the mean lux readings of the ambient light from the mean lux readings obtained from the exercise. The corrected readings [Table 1] for each lighting scenario were used to quantify and standardize the illumination from the chosen light sources. The images from the two apps were set to scale in the ImageJ software using the ruler captured in all images [Figure 2]. Subsequently, bleb area was annotated on the scaled images inside of ImageJ using the “free form selection” tool, twice by the senior ophthalmologist (PI). The annotation of the images was carried out on Microsoft Surface Pro 4 (Microsoft Inc.) device using the new Surface pen which has 4,000 levels of pressure sensitivity and 0.2 ms latency.

**Table 1:** Corrected illumination readings from the ocular surface using the Lux Light Meter Pro app for iPhone obtained by holding iPhone X at the focusing distance from the ocular surface


**Foot-candle (FC) readings using the Lux Light Meter Pro app for iPhone**			
**Illumination light source**	**Close**	**Intermediate**	**Distant**
**Yellow light**	301.392 lux (28 FC)	129.162 lux (12 FC)	43.056 lux (4 FC)
**White light**	290.628 lux (27 FC)	118.404 lux (11 FC)	32.292 lux (3 FC)

**Table 2:** Significance level (*p*-values) difference in bleb areas using ANOVA test in different lighting scenarios


**Scenarios** **Distance from the ocular surface**	**Close (4 cm) ** ***P*** **-value**	**Intermediate (7 cm) ** ***P*** **-value**	**Distant (12 cm) ** ***P*** **-value**
**WL F–YL F**	0.5691	0.6623	0.0460
**WL N–YL N**	0.7390	0.3525	0.4393
**WL N–YL F**	0.9911	0.9639	0.0559
WL F, white light filmic; YL F, yellow light filmic; WL N, white light native; YL N, yellow light native; WL F, white light filmic; YL N, yellow light native			

**Table 3:** EXIF data for the images obtained at the focusing distance from the Native camera app on iPhone X


**Camera parameters** **Illumination setting**	**Close illumination (4 cm)**	**Intermediate illumination (7 cm)**	**Distant illumination (12 cm)**
**ISO (absolute value)**	16–20	20–30	30–50
**Shutter-speed (in seconds)**	1/250–1/331	1/120–1/200	1/80–1/100

**Table 4:** SNR analysis summary for different lighting scenarios, namely Close, Intermediate, and Distant


**Parameter**	**Close**	**Intermediate**	**Distant**
**Mean**	1.261726	2.210459762	2.812866
**Standard error**	0.089813	0.123797842	0.138773
**Median**	1.187366	2.09632066	2.914794
**Standard deviation**	0.701462	0.958933961	1.065938
**Sample variance**	0.492049	0.919554341	1.136225
	**Difference in mean SNR**		
	Close-intermediate	Intermediate-distant	Close-distant
*P*-value	3.83E-13	5.93651E-06	4.37E-15
**Difference**	0.9	0.6	1.5

### Data Analysis

Data of bleb area from the images was compiled and indexed for different lighting scenarios in a Microsoft Excel data sheet. Differences in bleb area in white light and yellow light for the three distances between Filmic–Filmic images, Native–Native images, and Native–Filmic pro images were analyzed for all 30 patients with an objective of ensuring repeatability and reproducibility. One-way ANOVA was used to compare mean differences between the different scenarios, namely WL F–YL F, WL N–YL N, WL N–YL F at three different distances. This gives 8º of freedom and 261 residuals for 30 observations each. ANOVA test was run on the dataset for differences in measured bleb areas in different lighting scenarios of all 30 patients using R software and *p*-values for all scenarios elucidated in Table 2. Bland–Altman agreement test was used to assess agreement on bleb area measurement in different lighting scenarios between two graders and plots were compiled (Supplementary data).

### Accounting for Low Light and Image Noise

Signal-to-noise ratio (SNR) was chosen as the parameter of choice as an objective measure of image quality in terms of image noise due to low light.^[10]^ A reference image was chosen from images collected from a slit-lamp camera as this allowed comparison with the gold standard. As highlighted earlier, the images from the two different apps had slightly different resolutions and to prevent this from affecting results, the native app images were all cropped to match the resolution of images obtained from the slit-lamp camera. SNR was then calculated for all the images in the native camera app sample using the ImageJ software with the SNR plugin (written by Daniel Sage at the Biomedical Image Group, EPFL, Switzerland) wherein low SNR means higher noise than reference image and vice-versa.

### Accounting for Overexposure and Glaring

Manual evaluation of all patients' images in the three illumination settings were done by two experienced ophthalmologists (PI and ST) twice (in the second round the order of presentation of images was changed) and images were rated from Ex1 to Ex5. Ex1 represented gross overexposure where bleb morphology was completely obliterated; Ex2 represented mild overexposure wherein bleb morphology was somewhat retained; Ex3 represented the ideal exposure wherein all morphological features of the bleb were best assessed; Ex4 represented mild underexposure wherein bleb features were dark but visible; and Ex5 represented underexposure wherein bleb features were too dark to assess.

##  RESULTS 

The EXIF data from the images obtained using the native camera app varied over ranges [Table 3] instead of a specific value as the imaging parameters cannot be prefixed using the native camera app. Even though this was the case, the overall trend does reflect the changes in illumination as with decreasing illumination the ISO ranges increase and the shutter speed ranges decrease.^[11]^

The parameters on the FiLMiC Pro app were manually set for closest focusing distance, lowest possible ISO value of 22, and a fast shutter speed of 1/144 s. The minimum ISO setting possible on iPhone X was chosen as it ensured minimal noise in the image. A fast shutter speed was chosen as it allowed the images to be shot without motion blur in the three illumination settings.

The results of bleb area analysis reflect that there was no significant difference in measuring bleb area from the images obtained in the three illumination settings using both the apps. One-way ANOVA results indicate that there is no significant difference in bleb area measurement amongst groups as *p*-value > 0.05 and there is failure to reject null hypothesis. Class-wise significance was also calculated and is summarized in Table 1. Bland-Altman analysis for bleb area measurement between two graders (PI and GK) indicated that all measurements fell between +0.08 and –0.06 mm ${}^{2}$ of the mean bleb area. The plots are shown in Supplementary data.

Cohen's kappa coefficient for subjective rating was 0.7 for images with distant illumination using white light and 0.65 for images clicked with illumination at an intermediate distance using yellow light, suggesting significant agreement between the observers in these scenarios.

### Results from the SNR Analysis

SNR for images at various distances is summarized in Table 4. Images taken at close distance had significantly lower SNR compared to intermediate (SNR difference = 0.9, *p* < 0.001) and distant (SNR difference = 1.5, *p* < 0.001) illumination. There was no significant difference between intermediate and distant illumination (SNR difference = 0.6, *p* < 0.001).

##  DISCUSSION

With advances in smartphone technology, the application of smartphones in ophthalmology has increased manifold all around the world, especially in the developing countries due to limited access to more sophisticated equipment such as slit-lamp camera, anterior segment OCT, or other complex imaging methods.^[7,12,13,14]^ Applications in telemedicine, tele-teaching, and archiving have become much more accessible as a result of upcoming smartphone-based photography techniques that enable a reliable examination of different parts of the eye.^[7,12,13,14]^ The native camera app on iPhone has automatic exposure metering wherein it self-adjusts the image parameters such as shutter-speed, ISO etc., to compensate for decreasing illumination. We know that the digital camera sensors are able to achieve the best image quality at their native ISO level (lowest ISO setting).^[11]^ Same holds true for the 1.22 um 12 MP PDAF sensor on iPhone X with OIS (Optical Image Stabilization). Automatic exposure metering on the native app increases ISO levels and decreases the shutter speed for decrease in scenic illumination. This results in loss in image quality, substantial increase in image noise, and introduction of motion blur. The image quality on iPhone X, with its newer optics and A11 Bionic processor, showed high level of compensation for changing illumination especially when using the tap to focus and exposure box. However, as per the Newton's inverse square law, intensity of electromagnetic waves, emitted from a point source, is inversely proportional to the squared distance from the point source.^[15]^


$$ℎ\propto 1/{}^{𝑅2},$$

where R is the distance of the given point from the point source.

Bleb area measurement did not show statistically significant difference between different lighting scenarios. This establishes good consistency of smartphone-based bleb photography using illumination settings presented in this study. Bland-Altman analysis indicated good inter-operator reliability thereby establishing reproducibility of the results. This is particularly relevant for bleb morphology assessment as these images may be reliably used for partial IBAGS classification or Wuerzburg classification of bleb morphology.^[16,17,18]^

SNR measures of native app images in different illumination settings clearly reflect the decreasing illumination as introduction of image noise is evident when illumination distance increases. Also, the qualitative rating scale of the Filmic pro app images demonstrates that image characteristics needed for bleb assessment do vary with changing illumination. Our general observation was that the best results (high SNR and Ex: 2–4) were obtained from intermediate illumination settings (12 FC) as there was adequate illumination to prevent high-ISO levels while not exceeding the threshold to have caused glaring and overexposure.

Glare noted in 100/360 (28%) images under close illumination and underexposure seen in 30/360 (8%) images under distant illumination can be explained by the exponential increase in light intensity at the ocular surface by reducing the distance and vice-versa, respectively, in accordance to the inverse square law. Therefore, we suggest that using the high shutter-speed setting can help expose correctly the overexposed area but it is harder to achieve uniform exposure of the entire field of interest as the area closer to the light source is heavily overexposed and the area slightly away is in shadow. This was noted in the close illumination native app images as automatic exposure metering results in higher shutter-speeds and gross underexposure of the shadow areas of the globe. The restricted dynamic range of smartphone cameras is an understandable reason for this limitation.^[19]^ There are built-in tools like HDR mode (High Dynamic Range mode) that use advanced software processing to try to preserve details in highlights and shadows to overcome this limitation in exposure metering. However, there is also a considerable fall in SNR with this due to exposure bracketing and therefore for our study we left this mode off. Using high ISO, as the native app does automatically and FiLMiC Pro does manually, may help achieve adequate exposure even in low light but this introduces digital noise in the image and thereby impedes complete assessment of the morphological features of the area of interest. Small sensor size and processing within smartphones is an understandable reason for this limitation.^[19]^

On the Filmic Pro app, we were able to demonstrate marked differences in illumination with our image sets as we had fixed all camera parameters to predecided values (ISO: 22 and shutter-speed: 1/144) allowing for no automatic exposure compensation. The use of a third-party app that allows manual control of all camera parameters on iPhone helped us overcome automatic exposure metering encountered on the native camera app, therefore allowing us to illustrate radical differences in image exposure with change in the intensity of incident ocular light. The more precise control of focus point, a very high shutter-speed, and a fixed low ISO setting achieved using this third-party app enabled the highest-quality images without motion blur with only variables being the illumination setting. The best results obtained were dramatically demonstrated with illumination at intermediate illumination range than from close or distant illumination where we observed overexposure or underexposure of the area of interest, respectively.

SNR is meaningless unless put in context using reference images. This application of SNR as a quality metric has been implemented in the past for both image- and video-quality assessment.^[20,21,22]^ The SNR analysis in this study involved use of reference images from slit-lamp camera for comparison. This enabled comparison between image quality varying distance in different lighting scenarios. SNR for the close illumination setting was significantly lower than other settings. This can be explained by the small zone of illumination in this setting that results in a large part of image receiving little to no light. More distanced illumination setting makes a more diffuse zone of illumination thereby resulting in more uniformly illuminated image hence the better SNR.

There were limitations to the current study. Images obtained from the Filmic Pro app and the native iPhone camera app had different native resolutions. This poses challenges that need processing like image resizing, binning, and scaling to enable comparisons. SNR calculated for native app images in this study used images from a slit-lamp camera as reference. This enabled comparison of images obtained with iPhone amongst themselves but posed challenges for comparing iPhone images to slit-lamp images. SNR was not calculated for FilMic Pro images as those were captured stills from 4k video images. A third-party app that captures images at native resolution would perhaps be better suited for comparison.

In summary, anterior segment photography using the newer iPhones has been successfully implemented for assessing trabeculectomy bleb morphology. Using a third-party camera application provides added control over the image parameters and helps restrict the image noise although bleb area measurement did not have any statistically significant difference from the native camera application. Ideal lighting is essential for ensuring optimal image quality and we found that distance illumination had the best SNR. Our subjective rating analysis indicated that distance illumination with white light is the ideal illumination for classification of bleb images. High SNR was highly associated with better subjective rating from our graders. Future research is warranted to expand upon utility of this imaging technique for illustrating a variety of bleb morphologies for a larger group of patients.

Smartphones have provided telemedicine opportunities that were not available in the past. With newer advances in camera technology in these mobile devices, high-resolution ocular imaging can be achieved. Further research to develop better-quality metrics for standardization of testing is needed for these images.

##  Financial Support and Sponsorship

Nil.

##  Conflicts of interest

There are no conflicts of interest.
